# Functionalization of the transition metal oxides FeO, CoO, and NiO with alkali metal atoms decreases their ionization potentials by 3–5 eV

**DOI:** 10.1007/s00894-018-3901-7

**Published:** 2019-01-05

**Authors:** Dawid Faron, Piotr Skurski, Iwona Anusiewicz

**Affiliations:** 1 Laboratory of Quantum Chemistry, Faculty of Chemistry, University of Gdańsk, Wita Stwosza 63, 80-308 Gdańsk, Poland; 2 Department of Technical Physics and Applied Mathematics, Gdańsk University of Technology, Narutowicza 11/12, 80-233 Gdańsk, Poland

**Keywords:** Mixed oxides, Superalkalis, Strong reducers, Transition metal oxides

## Abstract

The existence and stabilities of various neutral metal oxides of formula MO*N* and MO*N*_2_ (M = Fe, Co, Ni; *N* = Li, Na) and their corresponding cations MO*N*^+^ and MO*N*_2_^+^ are predicted using density functional theory (B3LYP) with the 6-311 + G(d) basis set. Ab initio calculations carried out at the CCSD(T)/6-311 + G(3df) level of theory reveal that the ionization potentials (IPs) of the oxides MO decrease by ca. 3–5 eV upon functionalization with *N* to give either MO*N* or MO*N*_2_. The influences of the chemical constitution and local spin magnetic moment (on the transition metal atom) of the oxide or cation on its IP are presented and discussed.

## Introduction

It is well known that modern computational chemistry utilizing high-performance computers and quantum chemistry software packages is used to analyze the information obtained from chemical experiments and to design new molecular systems with unusual geometries and coordination types that often contradict classical structural theories. The many quantum chemistry programs that are now available (following almost 50 years of continuous development in this field) and recent advances in visualization software have led to a rapid increase in the number of reports on new (non-classical) molecules with desired properties. Examples of such theoretical predictions include easy-to-build molecules whose structures are based on the concepts of hypercoordination and/or hypervalency [1–10]. A hypercoordinated molecule is a compound containing one or more atoms (or any univalent ligand) that exceed(s) typical valence expectations. On the other hand, it is well documented that adding an element to any neutral closed-shell molecule is a convenient way of altering some of its physicochemical properties, such as its electron affinity, polarizability, or reducing power [11–14]. For example, the addition of a halogen atom (X) to an alkali metal halide (MX) leads to the formation of a strong oxidizer (MX_2_). Daughter anions of such oxidizers (so-called superhalogen anions) are known to strongly bind an excess electron (the resulting excess electron binding energy of the superhalogen anion exceeds that of the halogen atoms X) [11]. On the other hand, the addition of a second alkali metal atom M to MX causes the formation of the hypermetalated molecule M_2_X with strong reducing properties (stronger than those of the M atom) [13]. It is also worth mentioning that theoretical predictions regarding the stabilities of many of these compounds have been confirmed by subsequent experiments [6, 9, 15, 16]. Hence, it seems that computational investigations of novel superhalogen anions or hypermetalated molecules can provide important results. It should also be noted that superhalogen molecules are crucial constituents of ionic liquids [17] and superacids [18], whereas hypermetalated species are often used in superionic conductors applied during the production of next-generation high-energy batteries [19].

In our previous paper, we reported findings concerning the possible existence of a series of triatomic oxides MO*N* (where M = Be, Mg, Ca; *N* = Li, Na, K) [20]. In these MO*N* systems, the alkali metal atom *N* is bound to the neutral closed-shell alkaline earth metal oxide (BeO, MgO, or CaO) via the oxygen atom, so the resulting mixed oxides can be considered to contain hypercoordinated oxygen. According to our findings, all of those MO*N* molecules are thermodynamically stable and characterized by ionization energies that are significantly smaller (by 2–3 eV) than those of their parent (i.e., unmodified) compounds MO [20].

In this contribution, we present the results of our theoretical investigations of the structures and properties of another group of mixed oxides (MO*N*) consisting of transition metal oxides (FeO, CoO, and NiO) functionalized through the attachment of an alkali metal atom (*N* = Li or Na). In addition, we determined whether even larger molecules with two alkali metal atoms attached to the metal oxide (MO*N*_2_) are geometrically and thermodynamically stable, and how their physicochemical properties change upon functionalization, i.e., in comparison to the corresponding MO and MO*N* molecules. Hence, our goals were to test the electronic and thermodynamic stabilities of hitherto unknown MO*N* and MO*N*_2_ systems that exhibit various total spin angular momentum values and to predict the ionization potentials (IPs) of these compounds, which we view as the products obtained through the attachment of one or two alkali metal atoms to the transition metal oxides FeO, CoO, and NiO.

## Methods

The equilibrium structures of the molecules and cations MO*N*, MO*N*_*2*_, MO*N*^+^, and MO*N*_2_^+^ (where M = Fe, Co, Ni; *N* = Li, Na) and the harmonic vibrational frequencies of their minimum-energy structures (corresponding to various spin multiplicities) were calculated by applying Becke’s three-parameter hybrid method with the LYP (Lee–Yang–Parr) correlation functional (B3LYP) [21, 22] and the 6-311 + G(d) basis set [23]. The coupled-cluster method with single, double, and non-iterative inclusion of triple excitations (CCSD(T)) [24] and the enlarged 6-311 + G(3df) basis set were then used to calculate the final energies of the species at the geometries obtained with the B3LYP method.

Adiabatic ionization potentials (AIPs) were calculated as the energy difference between the neutral molecule (at its ground-state optimized geometry) and the corresponding cation (at its equilibrium structure).

Partial atomic charges and local spin magnetic moments on atoms were obtained from natural atomic orbital populations (NAO) calculated using the natural bond orbital (NBO) analysis scheme [25–29].

In order to avoid erroneous results arising from the implementation of default SCF calculations, the keyword SCF = NoVarAcc was used and two-electron integrals were evaluated (without prescreening) to a tolerance of 10^−20^ a.u. A convergence of 10^−8^ a.u. was used for the RMS density, while the convergence criterion for energy was set to 10^−7^ a.u.

The default cutoff values (as implemented in Gaussian16) for forces and step size, which determine the convergence, were used during the geometry optimization procedures employed. This means that the RMS force criterion was set to 3 × 10^−4^ a.u., whereas the maximum size for an optimization step (the initial trust radius) was set to 0.30 bohr or radians.

The local spin magnetic moment (obtained from NAO populations based on the NBO scheme) was calculated by including the Pop = NBORead keyword in the input file together with the $NBO AONLMO $END card, and was then extracted from the output by calculating the difference between the number of the electrons in the α and β spin representations.

The input files for all the calculations were prepared using the Molden [30] program, which generated either Cartesian or internal coordinates corresponding to the initial structures. The appropriate sets of Gaussian16 keywords and IOp values were then added to the input file to ensure that the desired calculations were performed. In particular, the keyword list included (aside from the method and basis set specifications) SCF = (NoVarAcc, Tight, IntRep), GFInput, Opt (or Freq), Pop = NBORead, and IOp(6/7 = 3).

All calculations were performed with Gaussian16 (rev. A.03) [31], while plots showing molecular structures were generated using the ChemCraft software [32].

## Results

### Testing the theoretical treatment applied

In order to determine the usefulness and accuracy of the theoretical treatment applied to calculate the ionization potentials of FeO*N*, CoO*N*, NiO*N*, FeO*N*_2_, CoO*N*_2_, and NiO*N*_2_ (*N* = Li, Na), we decided to perform several test calculations on the unmodified FeO, CoO, and NiO metal oxides, as the basic physicochemical properties of these oxides are well documented in the literature. In particular, our goal was to verify whether the vertical (VIP) and adiabatic (AIP) ionization potentials of FeO, CoO, and NiO determined at the level of theory applied in this contribution agreed with experimentally measured IPs reported previously [33, 34].

**Fig. 1:**
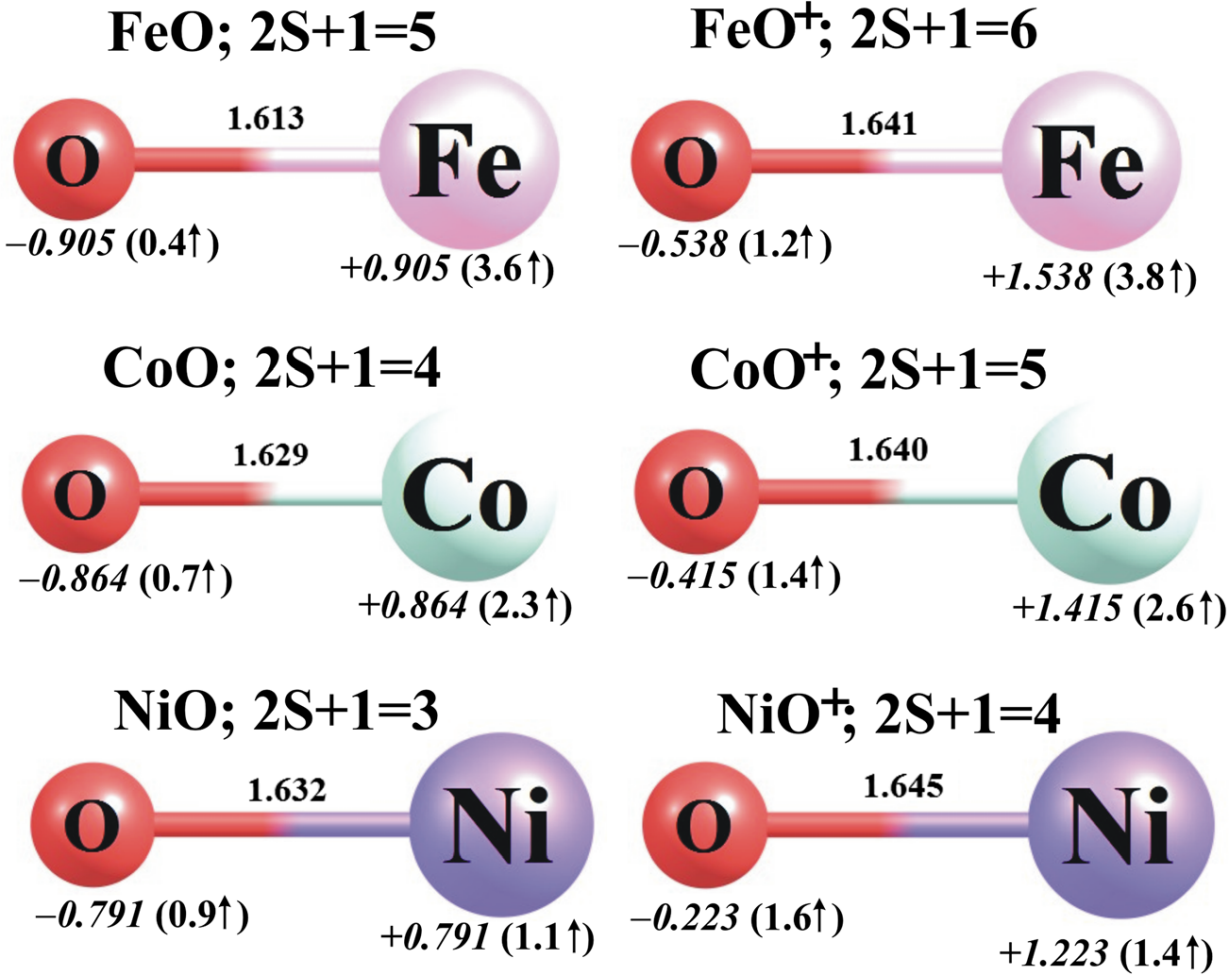
The B3LYP/6-311 + G(d) ground-state equilibrium structures (bond lengths in Å) of MO (*left column*) and MO^+^ (*right column*) molecules (M = Fe, Co, Ni), together with their partial atomic NBO charges in *e* (in *italics*) and local spin magnetic moments in μ_B_ (in *parentheses*)

The equilibrium structures of the neutral FeO, CoO, and NiO molecules and their corresponding cations are depicted in Fig. 1, while their VIPs and AIPs are collected in Table 1, where the experimental values are also provided for comparison. Since the changes in polarity that occur when the molecules of interest are functionalized are discussed in subsequent sections, we also provide experimental and computationally evaluated dipole moments (*μ*) of the neutral FeO, CoO, and NiO compounds in Table 1. According to our calculations (based on B3LYP/6-311 + G(d) geometry optimization followed by CCSD(T)/6-311 + G(3df) single point energy evaluation), the lowest-energy electronic states of the neutral FeO, CoO, and NiO correspond to the quintet, quartet, and triplet states, respectively, whereas those of the FeO^+^, CoO^+^, and NiO^+^ cations correspond to the sextet, quintet, and quartet states, respectively. Since these findings are in agreement with previous theoretical and experimental results [33–37], we feel confident that the theoretical treatment applied in this work is sufficient to allow the accurate characterization of the spin multiplicities of the lowest-energy electronic states of MO*N*, MO*N*_2_, MO*N*^+^, and MO*N*_2_^+^ (M = Fe, Co, Ni; *N* = Li, Na).

**Table 1:** The adiabatic ionization potentials (AIP, in eV) of MO (M = Fe, Co, Ni) molecules calculated using their structures optimized at the B3LYP/6-311 + G(d) level and their electronic energies obtained at the CCSD(T)/6-311 + G(3df) level of theory. The dipole moments (*μ*) are given in debyes. The experimental results (AIP^exp^ and *μ*^exp^) are taken from [33, 34]

Species	AIP^exp^	AIP/AIP*^(B3LYP)^	AIP/AIP*^(CCSD(T))^	*μ* ^exp^	*μ*
FeO	8.56 ± 0.01	8.860/9.458	8.507/8.940	4.5 ± 0.03	5.270
CoO	8.69 ± 0.2	8.769/10.120	8.355/9.335	4.5 ± 0.1	4.679
NiO	8.77 ± 0.18	9.270/10.930	8.964/10.010	4.5 ± 0.2	4.746

Analysis of the ionization potentials calculated at the CCSD(T)/6-311 + G(3df)//B3LYP/6-311 + G(d) level indicates that the AIPs obtained as the difference between the equilibrium ground-state energies of the neutral and cationic metal oxides are in satisfactory agreement with the experimental results: the values obtained for FeO (8.51 eV), CoO (8.36 eV), and NiO (8.96 eV) differ from the corresponding measured IPs by 0.05, 0.34, and 0.19 eV, respectively (see Table 1). On the other hand, the AIPs calculated at the B3LYP/6-311 + G(d) level (i.e., without the refinement of the final energies achieved by using the CCSD(T) method) are always overestimated (by up to 0.5 eV in the case of the NiO molecule) and seem to be less reliable (see Table 1). In addition, we provide the AIP*s, which we define as the ionization potentials obtained as the difference in energy between the ground-state neutral FeO, CoO, or NiO molecule at its optimized geometry and the corresponding low-spin cation (i.e., quartet FeO^+^, triplet CoO^+^, or doublet NiO^+^) at its equilibrium geometry. These calculated AIP* values were found to be 0.38–2.16 eV higher than the experimental IPs; see Table 1. Therefore, in the following section describing the adiabatic ionization potentials of MO*N* and MO*N*_2_ molecules, we consider the AIPs calculated at the CCSD(T)/6-311 + G(3df)//B3LYP/6-311 + G(d) level to be the most reliable, and we limit our discussion to those results; the AIPs obtained at the B3LYP/6-311 + G(d) level and the AIP* values are provided for comparison only.

### MO*N* and MO*N*^+^ systems (M = Fe, Co, Ni; *N* = Li, Na)

**Fig. 2:**
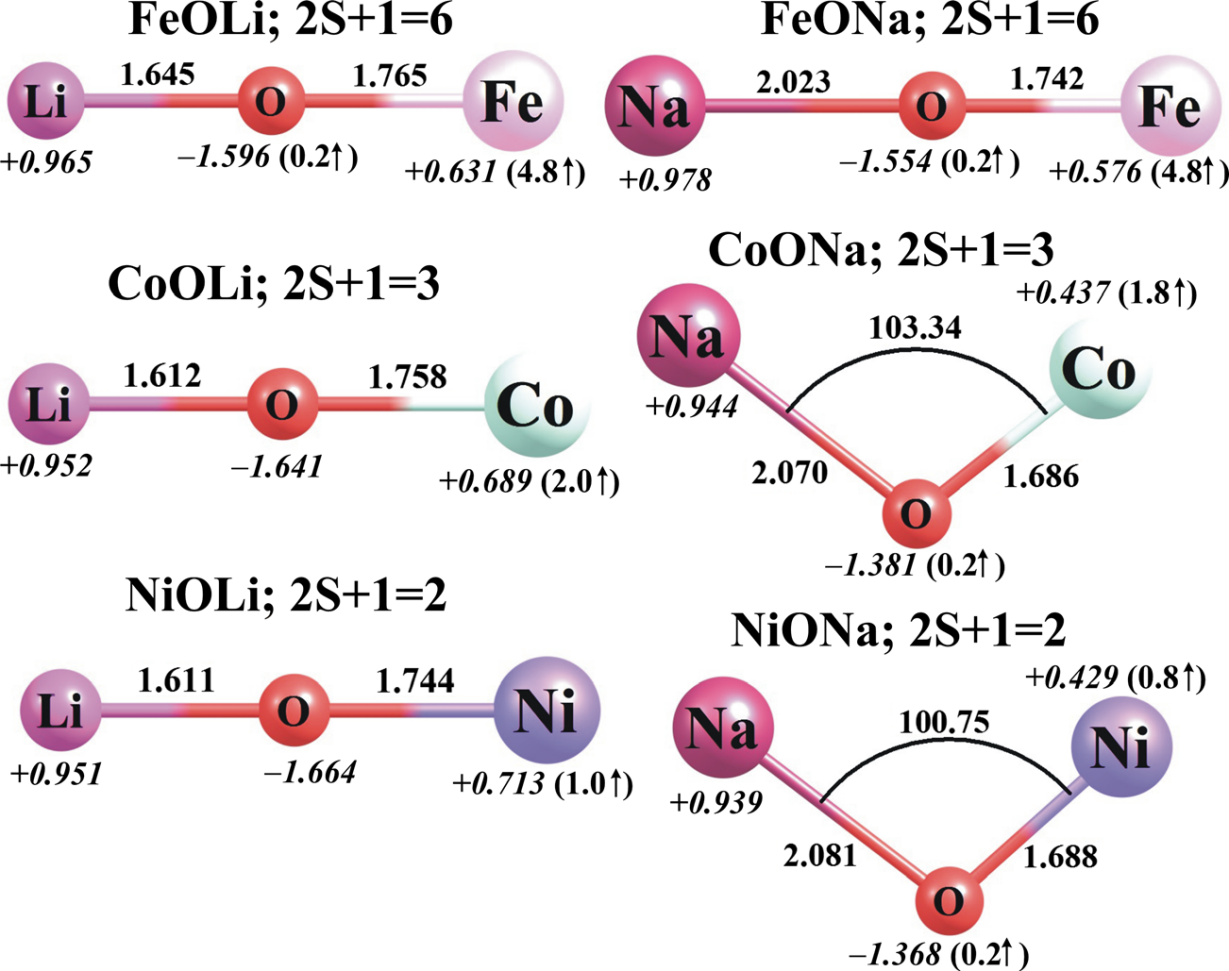
The ground-state equilibrium structures (bond lengths in Å) of the neutral MO*N* species (M = Fe, Co, Ni; *N* = Li, Na) calculated at the B3LYP/6-311 + G(d) level, together with their partial atomic NBO charges in *e* (in *italics*) and local spin magnetic moments in μ_B_ (in *parentheses*)

**Fig. 3:**
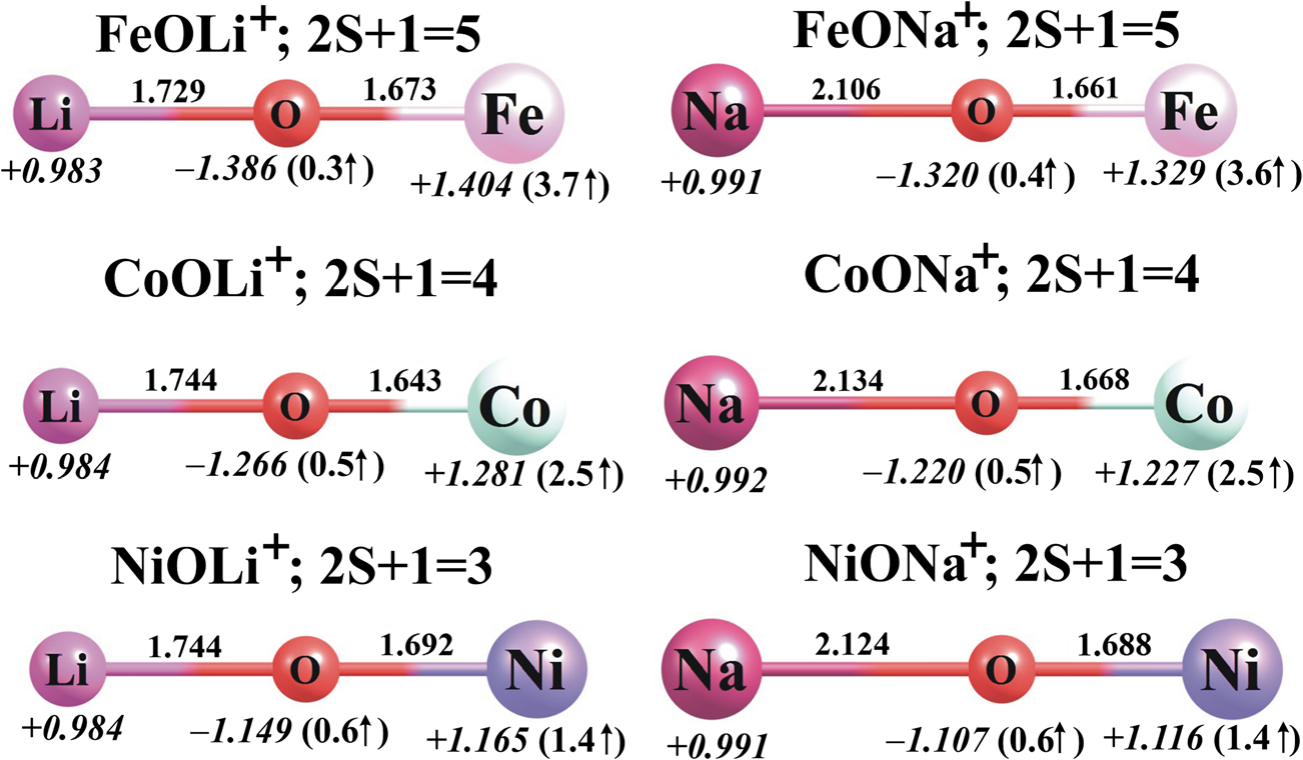
The ground-state equilibrium structures (bond lengths in Å) of the cationic MO*N*^+^ species (M = Fe, Co, Ni; *N* = Li, Na) calculated at the B3LYP/6-311 + G(d) level, together with their partial atomic NBO charges in *e* (in *italics*) and local spin magnetic moments in μ_B_ (in *parentheses*)

The lowest-energy MO*N* and MO*N*^+^ (M = Fe, Co, Ni; *N* = Li, Na) structures predicted at the B3LYP/6-311 + G(d) level are shown in Figs. 2 and 3, while their ionization potentials, dipole moments (*μ*), and harmonic vibrational frequencies are collected in Table 2.

**Table 2:** The adiabatic ionization potentials (AIP in eV) of MO*N* and MO*N*_2_ (M = Fe, Co, Ni; *N* = Li, Na) molecules, as calculated using the structures optimized at the B3LYP/6-311 + G(d) level and the electronic energies obtained at the CCSD(T)/6-311 + G(3df) level of theory. Dipole moments (*μ*) are given in debyes. Unscaled B3LYP harmonic vibrational frequencies (in cm^−1^) are also provided

Species	AIP/AIP*^(B3LYP)^	AIP/AIP*^(CCSD(T))^	*μ*	Vibrational frequencies
FeOLi	5.858/−	5.881/−	6.450	*ν*_1,2_ = 110 (*π*) *ν*_3_ = 580 (*σ*) *ν*_4_ = 966 (*σ*)
CoOLi	5.967/7.459	5.527/7.087	3.008	*ν*_1,2_ = 113 (*π*) *ν*_3_ = 539 (*σ*) *ν*_4_ = 993 (*σ*)
NiOLi	6.357/7.779	6.162/7.192	2.668	*ν*_1,2_ = 115 (*π*) *ν*_3_ = 542 (*σ*) *ν*_4_ = 997 (*σ*)
FeONa	5.392/−	5.376/−	7.837	*ν*_1,2_ = 79 (*π*) *ν*_3_ = 339 (*σ*) *ν*_4_ = 807 (*σ*)
CoONa	5.632/7.375	5.512/7.059	6.472	*ν*_1_ = 83 (*a’*) *ν*_2_ = 439 (*a’*) *ν*_3_ = 754 (*a’*)
NiONa	5.997/7.395	5.948/6.827	6.449	*ν*_1_ = 94 (*a’*) *ν*_2_ = 443 (*a’*) *ν*_3_ = 741 (*a’*)
FeOLi_2_	5.281/5.862	4.848/5.929	5.926	*ν*_1_ = 98 (*b*_1_) *ν*_2_ = 144 (*b*_2_) *ν*_3_ = 181 (*a*_1_) *ν*_4_ = 554 (*a*_1_) *ν*_5_ = 668 (*a*_1_) *ν*_6_ = 853 (*b*_2_)
CoOLi_2_	4.728/−	4.699/−	5.390	*ν*_1_ = 100 (*b*_1_) *ν*_2_ = 148 (*b*_2_) *ν*_3_ = 174 (*a*_1_) *ν*_4_ = 502 (*a*_1_) *ν*_5_ = 677 (*a*_1_) *ν*_6_ = 885 (*b*_2_)
NiOLi_2_	4.791/−	4.424/−	4.634	*ν*_1_ = 114 (*b*_1_) *ν*_2_ = 154 (*b*_2_) *ν*_3_ = 189 (*a*_1_) *ν*_4_ = 503 (*a*_1_) *ν*_5_ = 675 (*a*_1_) *ν*_6_ = 880 (*b*_2_)
FeONa_2_	4.864/5.674	4.438/5.638	5.905	*ν*_1_ = 69 (*b*_1_ ) *ν*_2_ = 91 (*a*_1_) *ν*_3_ = 101 (*b*_2_) *ν*_4_ = 294 (*a*_1_) *ν*_5_ = 515 (*b*_2_) *ν*_6_ = 620 (*a*_1_)
CoONa_2_	4.431/−	4.184/−	5.501	*ν*_1_ = 68 (*b*_1_) *ν*_2_ = 84 (*a*_1_) *ν*_3_ = 95 (*b*_2_) *ν*_4_ = 294 (*a*_1_) *ν*_5_ = 528 (*b*_2_) *ν*_6_ = 582 (*a*_1_)
NiONa_2_	4.394/−	3.960/−	4.704	*ν*_1_ = 71 (*b*_1_) *ν*_2_ = 92 (*a*_1_) *ν*_3_ = 107 (*b*_2_) *ν*_4_ = 292 (*a*_1_) *ν*_5_ = 527 (*b*_2_) *ν*_6_ = 582 (*a*_1_)

According to our findings, the lowest-energy isomers of all of the neutral MOLi molecules considered here have linear structures with *C*_*∞*v_ symmetry, while the lowest-energy isomers of all of the MONa systems are either linear (FeONa) or bent (CoONa and NiONa); see Fig. 2. We found that the linear structures of the CoONa and NiONa systems are not geometrically stable, and they each possess one (degenerate) imaginary frequency (26*i* and 15*i* cm^−1^, corresponding to the Co–O–Na and Ni–O–Na bending modes, respectively). As indicated in Figs. 2 and 3, the most stable FeO*N* molecules present high-spin (sextet) states, whereas the lowest-energy structures of the NiO*N* and CoO*N* compounds correspond to low-spin (doublet and triplet, respectively) states. We also observed that the high-spin (quartet and quintet) states of the NiO*N* and CoO*N* molecules at their linear geometries are higher in energy than their corresponding global minima (doublet and triplet states), respectively, by ca. 9–12 kcal/mol and 3–11 kcal/mol (depending on the *N* atom considered).

Since the MO*N* structures can be regarded as consisting of an alkali metal atom *N* (Li or Na) bound to a transition metal oxide MO (FeO, CoO or NiO), we compared the M–O bond lengths, NBO partial atomic charges, and local spin magnetic moments of the MO*N* species with those of the corresponding MO oxides. As shown in Figs. 1 and 2, introducing an *N* atom into any MO molecule always leads to the elongation of the M–O bond. In particular, the Fe–O, Co–O, and Ni–O bonds in FeO*N*, CoO*N*, and NiO*N* are longer than the corresponding M–O distances in FeO, CoO, and NiO by 0.129–0.152 Å, 0.057–0.129 Å, and 0.056–0.112 Å, respectively. Clearly, these bond elongations are the result of changes in the electron density distribution caused by the attachment of the alkali metal atom to the metal oxide.

The introduction of an alkali metal atom (Li or Na) into any of the FeO, CoO, and NiO molecules affects the electron density distribution in the metal oxide. NBO analysis of the MO*N* molecules indicates that the attachment of either Li or Na atoms to the MO system leads to a decrease in the positive charge (of 0.078–0.427 *e*) on the M atom with respect to the corresponding MO molecule, and causes the oxygen atom to become more negatively charged (see Figs. 1 and 2 for a comparison). As a result, the partial atomic charges on the MO subunit in each MO*N* system sum to ca. −1 *e* (i.e., −(0.939–0.978 *e*)), which corresponds to the positive charge on the attached alkali atom. Hence, each MO*N* system studied should be considered a strongly interacting MO^−^/*N*^+^ ionic pair. These changes in electron density distribution undoubtedly manifest themselves as differences in the predicted dipole moments of the MO molecules and their corresponding MO*N* compounds. Indeed, the dipole moment (*μ*) of FeO increases by 1.180 and 2.567 D when a Li or Na atom is attached, respectively (see Tables 1 and 2). As far as CoO and NiO systems are concerned, their polarities decrease and increase, respectively, upon the introduction of the alkali atom because the resulting MO*N* structures (M = Co, Ni) are linear for *N* = Li and bent for *N* = Na. Thus, adding a lithium atom to CoO or NiO results in a reduction in *μ* of 1.671 and 2.078 D, respectively, whereas adding a sodium atom to either CoO or NiO increases the dipole moment by 1.793 and 1.703 D, respectively.

Adding an alkali metal atom *N* to the metal oxides considered here also affects the local spin magnetic moments of the transition metal atoms (calculated as the difference between the number of the electrons in the α representation and the number of electrons in the β spin representation; values are presented in Figs. 1 and 2). In particular, the local spin magnetic moment on iron increases by 1.2 μ_B_ in FeO*N*, whereas the local spin magnetic moment on cobalt decreases by only 0.3–0.5 μ_B_ in CoO*N* and the local spin magnetic moment on nickel decreases by only 0.1–0.3 μ_B_ in NiO*N*.

Ionization of the MO*N* systems leads to the corresponding MO*N*^+^ cations, whose equilibrium structures were found to be linear (*C*_*∞*v_ symmetry) in all cases; see Fig. 3. Therefore, the geometries of the MO*N*^+^ cations are similar to those of their neutral parents except for CoONa^+^ and NiONa^+^, whose neutral parents are bent and exhibit *C*_s_ symmetry. Moreover, the M–O and O–*N* bonds change in length slightly following ionization: the M–O distances shorten by less than 0.11 Å while the *N*–O bonds elongate by 0.04–0.13 Å. As far as the multiplicities of the lowest-energy MO*N*^+^ systems are concerned, we found that the ground states of the FeO*N*^+^, CoO*N*^+^, and NiO*N*^+^ cations correspond to quintet, quartet, and triplet multiplicities, respectively. The nearest spin states (which have higher multiplicities in the cases of FeO*N*^+^ and CoO*N*^+^ and lower in the case of NiO*N*^+^) were found to be 32–39 kcal/mol higher in energy than the corresponding ground states. As shown in Figs. 2 and 3, the local spin magnetic moment on Fe decreases by 1.1–1.2 μ_B_, whereas the local spin magnetic moments on Co and Ni increase by 0.5–0.7 and 0.4–0.6 μ_B_, respectively, upon transitioning from the ground state of MO*N* to that of MO*N*^+^.

A comparison of the partial atomic charges calculated for the neutral and positively charged systems indicates that the MO*N* → MO*N*^+^ process causes a substantial increase (of +0.452–0.773 *e*) in the positive charge localized on the M atom, which approximately neutralizes the partial negative charge on the neighboring oxygen atom in the resulting cations; see Figs. 2 and 3. Since the positive charge assigned to the Li or Na atom remains close to +1.0 *e* in all MO*N*^+^ cations, one may conclude that the MO*N*^+^ cations resemble the corresponding FeO, CoO, and NiO oxides with a Li^+^ or Na^+^ ion attached. Considering that all of the MO*N*^+^ cations have been shown to be thermodynamically stable (accounting for the fragmentation processes that lead to the loss of the alkali metal ion; see the following section), our results indicate that the attachment of either Li^+^ or Na^+^ to any of the FeO, CoO, and NiO oxides should be a favorable process at room temperature.

The stability of each neutral MO*N* system was verified by calculating the Gibbs free energies (Δ*G*_298,r_) for two dissociation channels at *T* = 298.15 K: loss of the alkali metal atom *N* (MO*N *→ MO + *N*), and detachment of the transition metal atom M (MO*N* →* N*O + M). For each MO*N*^+^ cation, we considered three fragmentation paths: (i) MO*N*^+^ → MO + *N*^+^; (ii) MO*N*^+^ → * N*O + M^+^; and (iii) MO*N*^+^ → * N*O^+^+M. The positive Δ*G*_298,r_ values (which were within the ranges of 39–159 kcal/mol and 29–280 kcal/mol for the reactions involving MO*N* and MO*N*^+^, respectively) indicate that all of the MO*N* and MO*N*^+^ systems considered are indeed thermodynamically stable and not susceptible to fragmentation at room temperature.

As explained above, we view each MO*N* molecule as the MO oxide functionalized by the attachment of an alkali metal atom *N*. Hence, a comparison of the ionization potentials of the MO*N* species to those of the corresponding MO molecules allows us to establish the effects caused by this functionalization. According to our calculations, the AIP value decreases significantly upon the attachment of an alkali metal atom *N* to any of FeO, CoO, or NiO. In particular, the AIPs of the resulting functionalized oxides FeO*N* (5.88–5.38 eV), CoO*N* (5.53–5.51 eV), and NiO*N* (6.16–5.95 eV) were found to be ca. 2.6–3.1 eV lower than those of the corresponding unfunctionalized oxides (see the AIPs(CCSD(T)) values of the MO and MO*N* molecules gathered in Tables 1 and 2). Given that the AIPs of the unmodified (MO) metal oxides span the range 8.355–8.964 eV, the ionization potential decreases by ca. 31–37%, and must therefore be considered substantial.

### MO*N*_2_ and MO*N*_2_^+^ (M = Fe, Co, Ni; *N* = Li, Na)

**Fig. 4:**
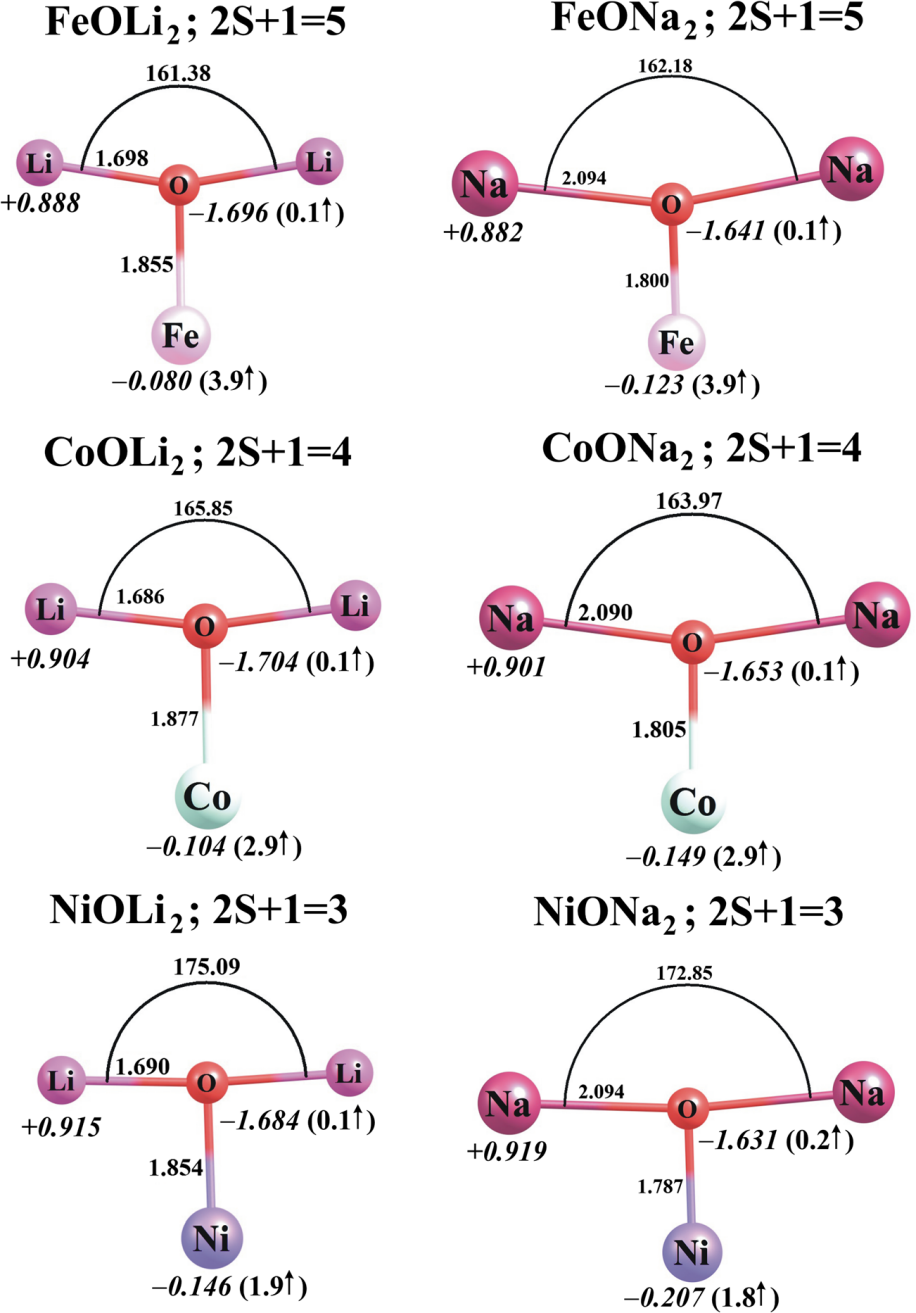
The ground-state equilibrium structures (bond lengths in Å) of the neutral MO*N*_2_ species (M = Fe, Co, Ni; *N* = Li, Na) calculated at the B3LYP/6-311 + G(d) level, together with their partial atomic NBO charges in *e* (in *italics*) and local spin magnetic moments in μ_B_ (in *parentheses*)

**Fig. 5:**
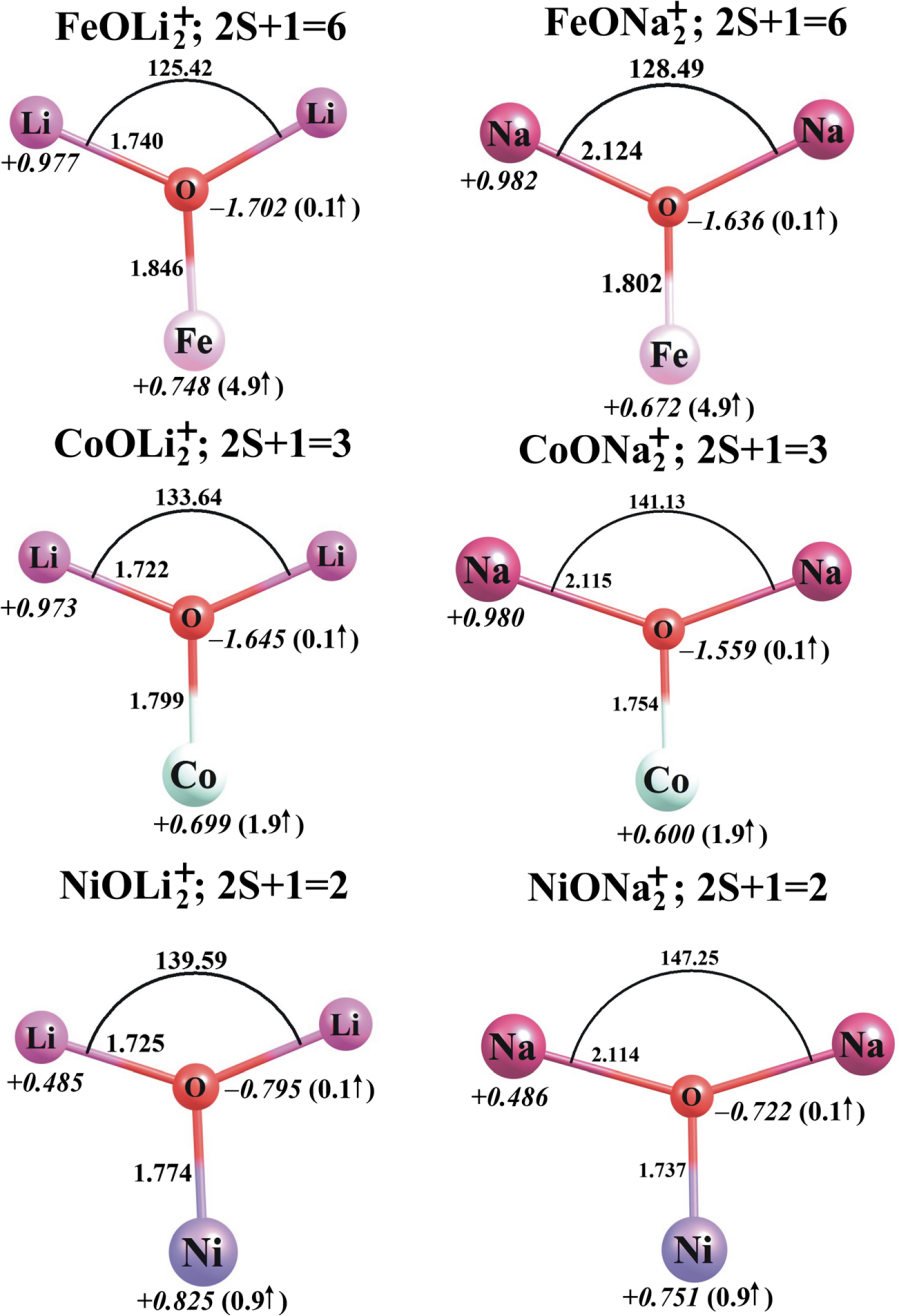
The ground-state equilibrium structures (bond lengths in Å) of the cationic MO*N*_2_^+^ species (M = Fe, Co, Ni; *N* = Li, Na) calculated at the B3LYP/6-311 + G(d) level, together with their partial atomic NBO charges in *e* (in *italics*) and local spin magnetic moments in μ_B_ (in *parentheses*)

The lowest-energy structures found for the neutral MO*N*_2_ molecules and their corresponding MO*N*_2_^+^ cations (M = Fe, Co, Ni; *N* = Li, Na) are depicted in Figs. 4 and 5, respectively, whereas the ionization potentials, dipole moments (*μ*), and harmonic vibrational frequencies of all the MO*N*_2_ systems considered here are collected in Table 2.

Our calculations revealed that the lowest-energy isomers of the neutral MO*N*_2_ species are of *C*_2v_ symmetry and correspond to deformed T-shaped structures with both alkali metal atoms bound to the oxygen atom (see Fig. 4). A comparison of the MO*N* and MO*N*_2_ structures indicates that the attachment of the second alkali metal atom to the MO*N* oxide leads to further, albeit small, increases in the M–O and *N*–O distances. In other words, the M–O and *N*–O bonds in MO*N*_2_ systems are longer than those in their corresponding MO*N* molecules by 0.06–0.12 and 0.01–0.08 Å, respectively.

The lowest-energy states of CoO*N*_2_ and NiO*N*_2_ possess multiplicities of four and three, respectively, whereas FeO*N*_2_ molecules are most stable with a multiplicity of five, which means that the lowest-energy states of CoO*N*_2_ and NiO*N*_2_ systems correspond to a higher total spin angular momentum quantum number *S* than for the corresponding MO*N* molecules, while those of FeO*N*_2_ correspond to a lower quantum number *S* than for the corresponding FeO*N* molecules. This increase or decrease in the multiplicity of the MO*N*_2_ molecule upon the attachment of the second alkali metal atom is consistent with the change in the local spin magnetic moment on the transition metal atom. In particular, the local spin magnetic moment on the iron in FeO*N*_2_ decreases by 0.9 μ_B_ in comparison to FeO*N*, whereas that on the cobalt in CoO*N*_2_ and that on the nickel in NiO*N*_2_ increase by 0.8–1.1 μ_B_ with respect to the corresponding values for CoO*N* and NiO*N*. It is also worth mentioning that the second lowest energy structures of FeO*N*_2_ and CoO*N*_2_ are of a higher multiplicity (seven and six, respectively) and are much higher in total energy (by ca. 15–29 kcal/mol as calculated at the B3LYP/6-311 + G(d) level) than the corresponding ground states. However, the singlet states obtained for NiOLi_2_ and NiONa_2_ are only ca. 3 and 5 kcal/mol higher in energy that their triplet states. This suggests that there is likely to be competition between the triplet and singlet states of NiO*N*_2_, even at room temperature.

Population analysis of the MO*N*_2_ molecules indicates that the attachment of the second alkali metal atom to a neutral MO*N* molecule leads to a significant reduction in the partial charge on the transition metal atom (of 0.586–0.859 *e*). As a consequence, the MO fragment becomes more negative (compared to the MO fragment in MO*N*), and its partial atomic charges sum to −(1.764–1.839 *e*), while the partial atomic charges on both alkali metal atoms are positive, ranging from +0.882 *e* to +0.919 *e*; see Figs. 2 and 4. The attachment of the second alkali metal atom to MO*N* molecules changes their electron density distributions, which in turn alters their dipole moments. Namely, FeOLi_2_ and FeONa_2_ are less polar (by 0.524 and 1.932 D, respectively) than the corresponding FeO*N* compounds, and a similar effect is predicted for CoONa_2_ and NiONa_2_, whose dipole moments are reduced (by 0.971 and 1.745 D, respectively) in comparison to CoONa and NiONa; see Table 2. On the contrary, the dipole moments of CoOLi_2_ and NiOLi_2_ are larger (by 2.382 and 1.966 D, respectively) than those of the corresponding systems containing only one alkali metal atom. Since the MO*N*_2_ molecules are structurally more similar to each other than the MO*N* compounds are (the structures of the latter are either linear or bent), their polarities are rather similar. Indeed, the dipole moments of all MO*N*_2_ molecules considered here span the range 4.634–5.926 D.

Ionization of the *C*_2v_-symmetry neutral MO*N*_2_ molecules leads to structurally analogous *C*_2v_-symmetry MO*N*_2_^+^ cations in which both alkali metal atoms remain bound to the oxygen atom (see Fig. 5). In fact, the loss of an electron from any of the neutral MO*N*_2_ species causes only small changes in the M–O and O–*N* bond lengths (<0.08 Å), whereas the *N*–O–*N* valence angle is strongly affected and its value decreases by ca. 23–36°. The lowest-energy states of the FeO*N*_2_^+^, CoO*N*_2_^+^, and NiO*N*_2_^+^ cations are sextet, triplet, and doublet states, respectively. The energetically closest spin states of the MO*N*_2_^+^ cations were found to lie 14–33 kcal/mol above their corresponding ground states. It is worth noting that the ground states of MO*N*_2_^+^ systems have the same multiplicities as the corresponding neutral MO*N* molecules, so they possess very similar local magnetic moments at their transition metal sites (see Figs. 3 and 5 for a comparison). This in turn means that the magnetic moment on Fe increases whereas those on Co and Ni decrease by about 1 μ_B_ upon the process MO*N*_2_ → MO*N*_2_^+^.

Analysis of differences in partial atomic charges for the FeO*N*_2_/FeO*N*_2_^+^ and CoO*N*_2_/CoO*N*_2_^+^ pairs indicates that ionization of FeO*N*_2_ or CoO*N*_2_ primarily involves a decrease in electron density at the transition metal atom. The partial atomic charge on Fe or Co becomes 0.749–0.828 *e* more positive, whereas the charges on the oxygen and alkali metal atoms change only slightly (by less than 0.1 *e*) with ionization; see Figs. 4 and 5 for comparison. In contrast, the change in the charge distribution is very different for the NiO*N*_2_ → NiO*N*_2_^+^ ionization process: there are significant increases in the partial charges on nickel (of 0.96–0.97 *e*) and oxygen (of 0.89–0.91 *e*) atoms, whereas the charges on the alkali metal atoms are reduced by ca. 0.43 *e* each; see Figs. 4 and 5.

As far as the thermodynamic stability of each neutral MO*N*_2_ system is concerned, we examined three possible dissociation channels: (i) loss of an alkali metal atom (MO*N*_2_ →  MO*N* + *N*), (ii) detachment of the *N*_2_ molecule (MO*N*_2_ → MO + *N*_2_), and (iii) fragmentation leading to the separation of the M atom from the *N*_2_O molecule (MO*N*_2_ → M + *N*_2_O). Similarly, we considered three reaction paths (i.e., MO*N*_2_^+^ → MO*N* + *N*^+^, MO*N*_2_ → MO + *N*_2_^+^, and MO*N*_2_ → M + *N*_2_O^+^) for each MO*N*_2_^+^ cation to check its thermodynamic stability. According to our estimates, the MO*N*_2_ compounds considered here and their daughter MO*N*_2_^+^ cations are stable to the fragmentation paths described above (as indicated by large positive Δ*G*_298,r_ values of 25–146 kcal/mol and 42–164 kcal/mol for the reactions involving MO*N*_2_ and MO*N*_2_^+^, respectively).

A comparison of the results collected in Tables 1 and 2 indicates that the ionization potentials of transition metal oxides of formula MO can be decreased markedly by attaching two alkali metal atoms (*N*_2_). In particular, the AIPs of the resulting FeO*N*_2_, CoO*N*_2_, and NiO*N*_2_ were found to be equal to 4.44–4.85 eV, 4.18–4.70 eV, and 3.96–4.42 eV, respectively, which means that they are 0.8–2.0 eV lower than those calculated for the corresponding FeO*N*, CoO*N*, and NiO*N* molecules. Hence, functionalization of the transition metal oxides by attaching two alkali metal atoms leads to major decreases in their ionization potentials (as the AIPs of the unmodified oxides MO are 3.6–5.0 eV larger than those of the MO*N*_2_). Finally, it should also be mentioned that all of the MO*N*_2_ molecules considered in this work exhibit superalkali characteristics, which means that their ionization potentials (3.96–4.85 eV) are smaller than the ionization potentials of Na (5.14 eV) and Li (5.39 eV).

## Summary

Based on our theoretical calculations, we postulate the existence and thermodynamic stability of a series of neutral MO*N* and MO*N*_2_ compounds (M = Fe, Co, Ni and *N* = Li, Na) and their corresponding MO*N*^+^ and MO*N*_2_^+^ cations. Computations performed at the CCSD(T)/6-311 + G(3df) level of theory on the structures optimized at the B3LYP/6-311 + G(d) level revealed that:

(i) Adding one or two Li or Na atoms to the transition metal oxides FeO, CoO, and NiO leads to the formation of thermodynamically stable molecules
(ii) The molecules FeOLi, FeOLi_2_, FeONa, FeONa_2_, CoOLi, CoOLi_2_, CoONa, CoONa_2_, FeOLi, FeOLi_2_, FeONa, and FeONa_2_ are characterized by remarkably small ionization potentials of 5.38–6.16 eV for MO*N* and 3.96–4.85 eV for MO*N*_2_
(iii) Comparison of the adiabatic ionization potentials of singly substituted systems (MO*N*) to those of the corresponding unmodified transition metal oxides MO indicates that the former are much lower (by 2.6–3.1 eV, which corresponds to 31–37% lower) than the latter
(iv) Attachment of a second alkali metal atom to MO*N* causes the ionization potential to drop even further, as the resulting compounds MO*N*_2_ have AIPs that are 3.6–5.0 eV smaller than those of the corresponding MO molecules
(v) Relatively large local spin magnetic moments of 0.8–4.9 μ_B_ at the transition metal are predicted when neutral or cationic transition metal oxides are decorated with one or two alkali metal atoms.
